# The causal relationship in hypertensive encephalopathy: mechanistic analysis of five specific circulating cytokines

**DOI:** 10.1515/med-2026-1412

**Published:** 2026-04-24

**Authors:** Pengfei Zhang, Yijia Huang, Haoying Li, Can Zhang, Zhiren Chen

**Affiliations:** School of Basic Medical Sciences, Jiangxi Medical College, Nanchang University, Nangchang, China; School of Queen Mary, Jiangxi Medical College, Nanchang University Nanchang, China; XuZhou Central Hospital, Xuzhou, China

**Keywords:** hypertensive encephalopathy, mendelian randomization, cytokines

## Abstract

**Objectives:**

To investigate the causal relationship between genetically predicted levels of five circulating cytokines – CXCL11, IL-2, IL-4, leukemia inhibitory factor (LIF), and neurotrophin-3 (NT-3) – and the risk of hypertensive encephalopathy using a two-sample Mendelian randomization (MR) approach.

**Methods:**

Single nucleotide polymorphisms (SNPs) strongly associated with each cytokine (p*<*5 × 10^−8^) were selected from publicly available GWAS data. Summary statistics for HE were obtained from the FinnGen biobank (endpoint: I9_HYPERTENC). MR analyses were conducted using inverse-variance weighted (IVW), MR-Egger, weighted median, and weighted mode methods. Sensitivity analyses included heterogeneity testing, MR-Egger intercept assessment, and leave-one-out analysis.

**Results:**

Genetically higher levels of IL-2 (OR=0.35, 95 % CI: 0.13–0.99, p*=*0.043), IL-4 (OR=0.50, 95 % CI: 0.30–0.85, p*=*0.010), and NT-3 (OR=0.46, 95 % CI: 0.28–0.75, p*=*0.0038) were significantly associated with lower risk of HE. CXCL11 showed a significant positive association with HE risk (OR=2.79, 95 % CI: 1.26–6.20, p*=*0.028). LIF displayed a non-significant trend toward protection (OR=0.43, p*=*0.060). Results were consistent across sensitivity analyses with no evidence of pleiotropy or heterogeneity.

**Conclusions:**

This Mendelian randomization study provides evidence that genetically determined levels of IL-2, IL-4, and NT-3 are protective against hypertensive encephalopathy, while CXCL11 is a risk factor. These cytokines may serve as potential biomarkers or therapeutic targets for HE.

## Introduction

Hypertensive encephalopathy (HE) is a life-threatening hypertensive emergency characterized by severe blood pressure elevation accompanied by neurological symptoms (such as seizures, confusion, or lethargy) due to acute cerebrovascular dysfunction and brain edema [1]. This condition represents an extreme form of target-organ damage resulting from hypertension. However, the underlying biological mechanisms that predispose hypertensive patients to develop encephalopathy remain incompletely understood. Emerging evidence implicates inflammation and immune dysregulation in the development of hypertension and its complications [2]. Hypertension is associated with a chronic low-grade inflammatory state in which activated immune cells release pro-inflammatory cytokines that contribute to vascular injury and end-organ damage [3]. In the central nervous system, *in situ* inflammatory responses have been observed during hypertensive crises – for example, cerebrospinal fluid levels of interleukin-6 (IL-6) can reach markedly elevated levels in patients with acute hypertensive encephalopathy (IL-6 ∼328 pg/mL, vs.<4 pg/mL normally) [4], suggesting a potential role for cytokine-mediated neuroinflammation in HE.

Several circulating cytokines are potential contributors to the inflammatory response. to hypertension-related vascular pathology. This study focuses on five inflammatory mediators – CXCL11, IL-2, IL-4, LIF, and NT-3 – chosen based on their known roles in immune responses and availability of genetic instruments. CXCL11 (C-X-C motif chemokine ligand 11) is an interferon‐inducible T cell chemoattractant involved in Th1-type immune responses. In contrast, IL-2 and IL-4 are cytokines central to lymphocyte function: IL-2 (interleukin-2) promotes T cell proliferation (including regulatory T cells at low concentrations), whereas IL-4 (interleukin-4) is a signature Th2 cytokine with anti-inflammatory and immunomodulatory effects. LIF (leukemia inhibitory factor) is a pleiotropic cytokine from the IL-6 family, with reported cardioprotective effects in acute stress settings [5] and immunosuppressive properties in inflammation [6]. NT-3 (neurotrophin-3) is a neurotrophic growth factor that supports neuronal survival; intriguingly, NT-3 levels have been found to increase in animal models of hypertension, hinting at a potential compensatory or pathologic role in high blood pressure contexts [7]. Given prior evidence linking immune signaling to hypertension, it is plausible that genetically determined differences in these specific cytokine levels could influence the risk of hypertensive encephalopathy [8], 9]. However, observational associations are prone to confounding and reverse causation, making it difficult to infer causality.

Mendelian randomization (MR) is an epidemiological approach that uses genetic variants as instrumental variables to assess the causal influence of a modifiable exposure (here, cytokine levels) on an outcome (HE) [10]. Because alleles are randomly assorted at conception, MR analyses are less susceptible to environmental confounders and can strengthen causal inference if key assumptions are met [11]. Recent large-scale genome-wide association studies (GWAS) have identified common genetic variants associated with circulating cytokine levels, enabling the use of these variants as instruments for two-sample bidirectional Mendelian randomization (two-sample MR) analyses [12]. In this study, we applied a two-sample MR design to investigate whether genetically elevated levels of CXCL11, IL-2, IL-4, LIF, and NT-3 have a causal effect on the risk of hypertensive encephalopathy. Therefore, we hypothesized that genetically predicted levels of these five cytokines are causally associated with the risk of hypertensive encephalopathy. This study aimed to investigate these potential causal relationships using a two-sample Mendelian randomization approach. We employed multiple MR methods and conducted extensive sensitivity analyses, including MR-Egger regression and weighted median approaches, to ensure the robustness of our findings. Our aim was to clarify the potential causal role of inflammation in HE and identify specific cytokine pathways that could be targets for preventive strategies or therapeutic interventions.

## Methods

### MR assumptions

**Figure 1:**
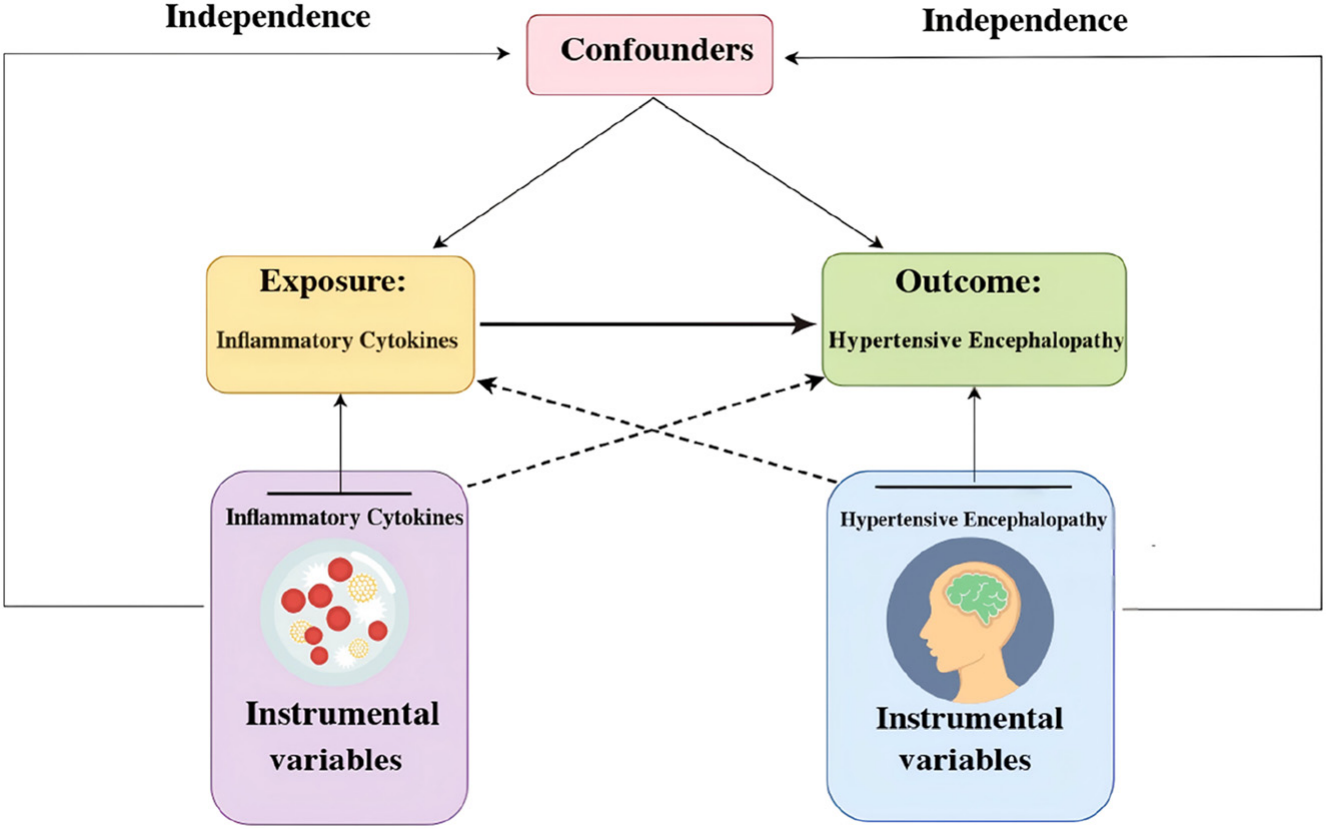
Schematic of the study design in this MR analysis.

In MR analyses, three fundamental assumptions must be satisfied: relevance, independence, and exclusion restriction [13]. Specifically, the selected genetic variants should be strongly associated with the exposure of interest (relevance), remain unaffected by any confounders in the exposure–outcome relationship (independence), and influence the outcome solely through the exposure rather than through any alternative pathways (exclusion restriction). In this study, we integrated data from two GWASs to identify genetic variants (SNPs) significantly associated with 91 inflammatory cytokines and hypertensive encephalopathy (Figure 1).

### Data source

We obtained summary statistics for inflammatory cytokines from a recent meta-analysis of genome-wide association studies (GWAS), which assessed 91 plasma proteins using the Olink Target platform among 14,824 participants in a genome-wide protein quantitative trait loci (pQTL) analysis [14]. Data on hypertensive encephalopathy were derived from the FinnGen R10 cohort, comprising 98 cases and 374,631 controls [15]. As this study utilized publicly available summary-level data, no additional ethics approval or participant consent was required. Detailed information on the data sources used in this MR analysis is provided in Table 1.

**Table 1:** Details of the genome-wide association studies and datasets used in our analysis.

Data	Samplesize	Links for download	PMID
Inflammatory cytokines	14,824 participants	https://www.phpc.cam.ac.uk/ceu/proteins	37,563,310
Hypertensive encephalopathy	98 cases, 374,631 controls	https://r10.finngen.fi/pheno/I9_HYPERTENC	34,737,426

### Selection of instrumental variables

We employed a significance threshold of p<1.0 × 10ˆ−5 to select instrumental variables (IVs). To ensure genetic independence among loci, we applied a linkage disequilibrium (LD) threshold of Rˆ2<0.001 and a clumping distance of 10,000 kb using the “TwoSampleMR” package in conjunction with the 1,000 Genomes EUR reference data. For each trait, we retained only the single nucleotide polymorphism (SNP) with the lowest p-value for clumping. We then extracted relevant information, including effect alleles, effect sizes (β-values), standard errors, and p-values, to calculate the proportion of variance explained (Rˆ2) and the F-statistic to assess instrument strength. These calculations were based on the following formulas:

$$R2=2\times \text{MAF}\times \left(1-\text{MAF}\right)\times \beta 2\text{;}$$

$$F=R2\left(n-k-1\right)/k\left(1-R2\right)\text{,}$$

where MAF denotes the minor allele frequency, n is the sample size, and k is the number of IVs [16], 17].

### Statistical analysis

We employed several methods to investigate the potential causal relationships between inflammatory cytokines and hypertensive encephalopathy, including the fixed-effects and random-effects inverse-variance weighted (IVW) methods, weighted median, MR–Egger regression, weighted mode, and Simple Mode test. The IVW method served as our primary analytical approach because of its precise effect estimates and widespread use in MR analyses [18], [19], [20]. Initially, individual SNPs were evaluated using the Wald estimator and the Delta method to obtain ratio estimates. These estimates were then pooled to derive the main causal effect estimate [21]. We assessed heterogeneity among selected SNPs using Cochran’s Q test; if heterogeneity was detected (p<0.05), the random-effects IVW model was applied; otherwise, the fixed-effects IVW method was used [22].

To ensure robust associations and address pleiotropy and potentially invalid instruments, we performed additional sensitivity analyses. First, we applied the weighted median method, which can yield valid causal estimates even when fewer than 50 % of the instruments are valid [23]. Second, we conducted MR–Egger regression to evaluate horizontal pleiotropy by examining the intercept term; an intercept p-value <0.05 was indicative of possible horizontal pleiotropy [23], 24]. Lastly, we employed the MR-PRESSO test to detect outliers via a global heterogeneity test. Outliers were removed, and a corrected association was subsequently derived [25]. All associations between inflammatory cytokines and hypertensive encephalopathy were reported as odds ratios (ORs) with 95 % confidence intervals (CIs). Analyses were performed using R version 4.2.2 and the “MendelianRandomization,” “TwoSampleMR,” and “MR-PRESSO” packages.

### Ethical approval

This research utilized publicly available GWAS summary data and did not involve human or animal participants, thus exempting it from ethical review requirements.

## Results

**Figure 2:**
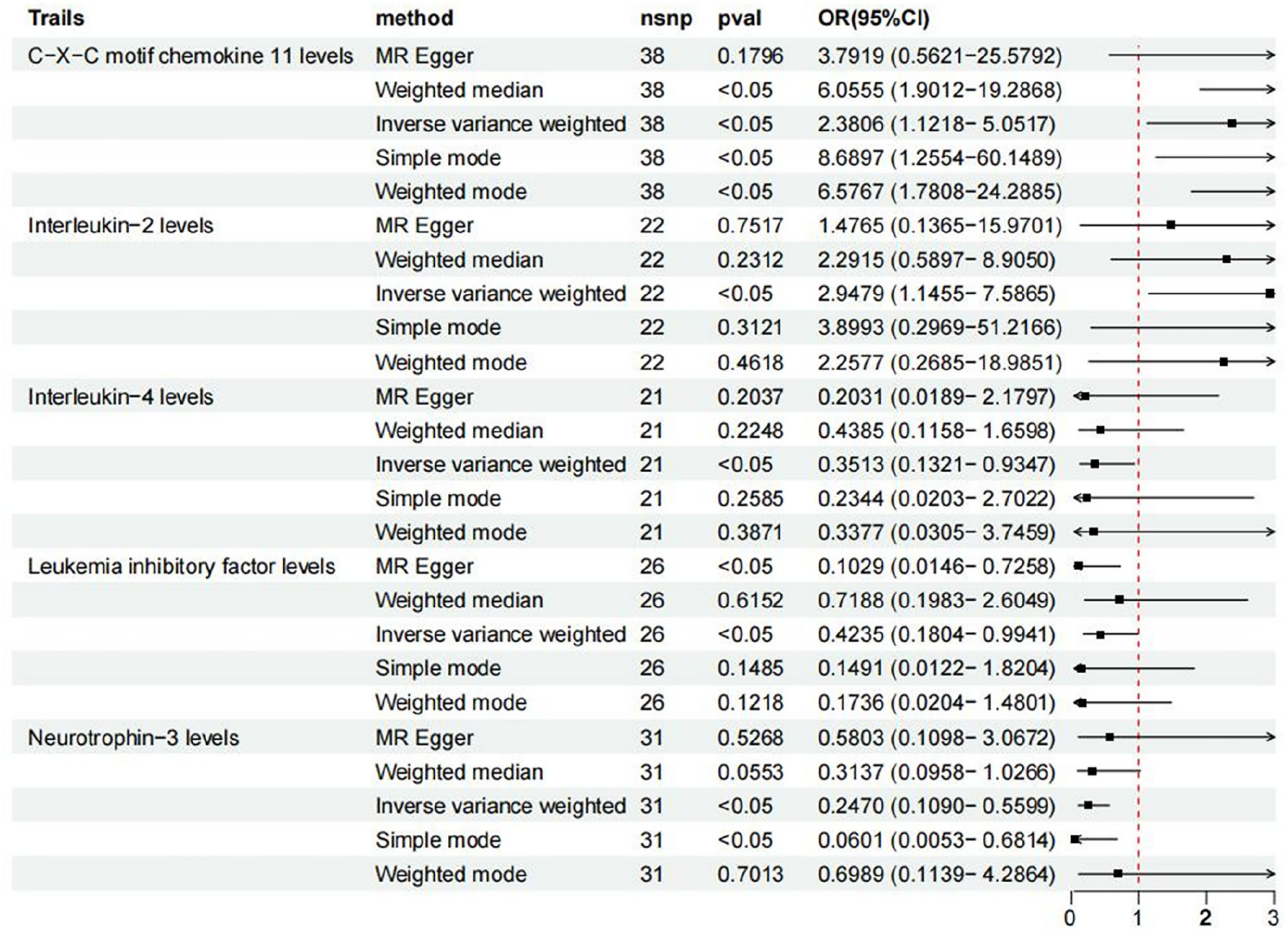
Forest plot of the associations between 5 genetically determined inflammatory cytokine traits and the risk of hypertensive encephalopathy.

We conducted a two-sample MR analysis using the inverse-variance weighted (IVW) method – our principal approach that combines genetic variant estimates weighted by their precision – to examine the potential causal relationship between inflammatory cytokines and hypertensive encephalopathy. Specifically, as illustrated in Figure 2, two inflammatory cytokines were associated with an increased risk of hypertensive encephalopathy.

### Heterogeneity analysis

The results of the Cochran Q test are shown in Table 2. For 5 inflammatory cytokines, the Cochran Q test did not detect any significant heterogeneity, thus a fixed-effect model was employed to estimate the MR Effect size.

**Table 2:** The results of the Cochran Q test for MR Egger and inverse variance weighted method. IVW: inverse variance weighted method.

Exposure	Outcome	Method	*Q*	df	p-Value
C−X−C motif chemokine 11	Hypertensive encephalopathy	MR Egger	37.466	36	0.402
		IVW	37.748	37	0.435
Interleukin−2		MR Egger	20.779	20	0.410
		IVW	21.180	21	0.448
Interleukin−4		MR Egger	12.239	19	0.875
		IVW	12.485	20	0.898
Leukemia inhibitory factor		MR Egger	19.002	24	0.752
		IVW	21.493	25	0.665
Neurotrophin−3		MR Egger	17.562	29	0.953
		IVW	18.895	30	0.942

### Mendelian randomization results

This MR study found that genetically predicted higher levels of Interleukin-2 (IL-2) (OR=0.35, 95 % CI: 0.13–0.99, p=0.043) was associated with a significantly decreased risk of hypertensive encephalopathy, whereas higher levels of C-X-C motif chemokine 11 (CXCL11) (OR=2.79, 95 % CI: 1.26–6.20, p=0.028) were associated with an increased risk. Conversely, genetically predicted higher levels of Leukemia inhibitory factor (LIF) (OR=0.43, 95 % CI: 0.18–0.99, p=0.060), Interleukin-4 (IL-4) (OR=0.50, 95 % CI: 0.30–0.85, p=0.010), and particularly Neurotrophin-3 (NT-3) (OR=0.46, 95 % CI: 0.28–0.75, p=0.0038) demonstrated a causal association with a significantly decreased risk of hypertensive encephalopathy, with the protective effect of NT-3 being the most pronounced.

## Discussion

In the present study, we found that genetically predicted higher levels of IL-2, IL-4, and NT-3 were associated with a lower risk of hypertensive encephalopathy, while CXCL11 was associated with an increased risk. Our findings provide novel evidence that specific immune mediators may modulate the development of this severe hypertensive complication. In particular, higher genetically predicted levels of IL-2, IL-4, and NT-3 were associated with a significantly lower risk of hypertensive encephalopathy, consistent with a protective effect, whereas higher levels of the chemokine CXCL11 were associated with higher HE risk, consistent with a harmful effect. The association for LIF was suggestive of a protective trend but did not reach statistical significance. To our knowledge, this is the first study to demonstrate these potential causal relationships using MR, leveraging genetic instruments to overcome confounding in assessing the role of inflammation in hypertensive emergencies.

IL-4 is a canonical Th2 cytokine that counter-regulates pro-inflammatory Th1 responses [26]. A shift toward a Th2-dominant immune profile – exemplified by elevated IL-4 – may attenuate the vascular inflammation and endothelial dysfunction that contribute to severe hypertension and encephalopathy. Notably, in preeclampsia, a condition characterized by placental ischemia-induced hypertension, exogenous IL-4 supplementation has been shown to reduce systemic inflammation, promote a Th2 response, and ultimately improve hypertension and end-organ outcomes [27]. This supports the concept that IL-4 exerts antihypertensive and end-organ protective effects in inflammatory hypertension. Our MR finding that genetically elevated IL-4 reduces HE risk aligns with this mechanism, suggesting that individuals with a genetic predisposition toward higher IL-4 levels may be partially protected from the extreme cerebral consequences of acute hypertension. Similarly, low-dose IL-2 is known to expand regulatory T cells and temper excessive immune activation [28]. Although IL-2 can exhibit dual roles – potentiating inflammation at high levels while enhancing immunoregulation at controlled concentrations – our finding of an inverse association between genetically predicted IL-2 and HE risk suggests that, on balance, lifelong higher IL-2 activity may foster a more regulated immune environment, possibly via Treg activity, thereby reducing the likelihood of acute hypertensive end-organ damage. This intriguing result warrants further investigation, as it highlights the IL-2/Treg axis as a potential modulator of severe hypertension outcomes.

The neurotrophic cytokine NT-3(neurotrophin-3) also emerged as a protective factor in our analysis. While NT-3 is predominantly recognized for its role in neuronal growth and repair [28], neurotrophins can also influence vascular and autonomic function. Previous studies have reported elevated NT-3 levels in spontaneously hypertensive rats [29], which may reflect a compensatory response to counteract hypertension-induced neural or vascular injury. Our MR results suggest that higher NT-3 levels causally lower the risk of hypertensive encephalopathy. We hypothesize that NT-3 may help preserve blood-brain barrier integrity or support cerebrovascular resilience during acute blood pressure surges, thereby preventing the cascade of events leading to encephalopathy. It is also conceivable that NT-3 influences autonomic nervous system regulation of blood pressure, although the precise pathways remain unclear. Regardless, this novel finding highlights NT-3 and its downstream signaling as important areas for future research. If confirmed, NT-3 could be explored as a biomarker for reduced susceptibility to hypertensive emergencies or as a therapeutic target for neuroprotection in severe hypertension.

This study is the first to use genetic instrumental variables to provide evidence for a causal relationship between specific cytokines and hypertensive encephalopathy. However, the biological mechanisms underlying the protective or detrimental effects we propose remain largely speculative. For instance, we hypothesize that IL-4 confers protection by promoting a Th2 immune shift, IL-2 by expanding regulatory T cells, and CXCL11 exacerbates neurovascular inflammation by recruiting Th1 lymphocytes. Although these hypotheses are consistent with established immunological and hypertensive pathophysiology, MR analysis itself cannot delineate the exact pathways of these molecules in specific processes such as blood-brain barrier integrity, cerebral blood flow autoregulation, or neuronal excitability. Similarly, while the protective association of NT-3 is notable, it remains unclear whether it acts by directly stabilizing cerebral vessels or through remote modulation of the autonomic nervous system during acute blood pressure elevation. Therefore, future studies must integrate experimental mechanistic investigations to validate and extend these genetic findings. Using animal models of hypertensive encephalopathy to intervene in specific cytokine pathways and evaluate effects on brain edema, neuroinflammatory markers, and clinical outcomes would provide direct biological validation. Furthermore, prospective cohort studies in patients with hypertensive emergencies, incorporating dynamic monitoring of these cytokine levels alongside neuroimaging and cerebrospinal fluid biomarkers, could help link genetic susceptibility with acute pathophysiological processes.

Notably, our study identified a potential protective trend for LIF (OR=0.43, p=0.060), which aligns with its known immunomodulatory and organ-protective properties. However, the statistical power of this association is limited by the relatively small number of HE cases, which may increase uncertainty in effect estimation and obscure a true weak-to-moderate association. Therefore, this non-significant trend underscores the need for confirmatory studies with larger sample sizes or alternative designs to clarify the exact role of LIF in hypertensive encephalopathy.

In contrast, CXCL11 appears to play a detrimental role. CXCL11 (also known as I-TAC) is an interferon-γ–induced chemokine that attracts activated T cells and NK cells via the CXCR3 receptor [30], thereby amplifying Th1-type inflammatory responses. Our MR analysis indicates that genetically higher CXCL11 levels substantially increase the risk of hypertensive encephalopathy. This finding is biologically plausible within the framework of pro-hypertensive immune mechanisms. Angiotensin II – a key mediator of hypertension – is known to promote a Th1 immune shift, increasing interferon-γ and CXCR3 chemokines while suppressing Th2 cytokines such as IL-4.

Previous research has implicated other inflammatory mediators, such as IL-6 and TNF-α, in hypertension and acute end-organ damage [31], [32], [33], [34]. While our study focused on five specific cytokines and revealed their potential causal roles in HE, this does not diminish the importance of other inflammatory mediators. Our findings suggest that the complex immune network in hypertensive encephalopathy may involve a delicate balance between specific pro-inflammatory and anti-inflammatory or neuroprotective signals. Future research, such as comprehensive proteomic MR analyses using a broader array of protein-related genetic instruments, could enable systematic screening of additional inflammatory mediators. Such an approach would provide a more complete map of immune drivers in hypertensive emergencies, potentially validating our findings and uncovering novel, previously overlooked pathogenic or protective pathways, thereby advancing a more integrated understanding of the immune system’s role in HE pathogenesis.

## Conclusions

In summary, this MR study provides evidence that circulating cytokine levels can causally influence the risk of hypertensive encephalopathy. Genetically higher levels of IL-4, IL-2, and NT-3 were associated with a substantially reduced risk of HE, suggesting that anti-inflammatory and neurotrophic pathways confer protection against the development of hypertensive brain edema. Conversely, higher levels of the pro-inflammatory chemokine CXCL11 increased the risk of HE, consistent with a detrimental role of Th1-mediated immune activation in hypertensive crises. These findings support the notion that the balance of the immune response – specifically, a tilt toward a Th2/regulatory profile vs. a Th1/pro-inflammatory profile – is an important factor in determining susceptibility to the most severe complications of hypertension. From a clinical standpoint, our results highlight novel potential biomarkers and therapeutic targets (such as IL-4/IL-2 augmentation or CXCL11 inhibition) for preventing hypertensive encephalopathy, although further research and validation are required. More broadly, this work demonstrates the utility of MR in unraveling the complex causal pathways between inflammation and cardiovascular emergencies. As large genetic datasets continue to grow, integrating genomic, proteomic, and clinical data will be a powerful approach to identify which inflammatory cascades are merely correlates and which are true contributors to hypertensive end-organ damage. Ultimately, a deeper understanding of these mechanisms could pave the way for immunomodulatory strategies to improve outcomes in patients with malignant hypertension and hypertensive encephalopathy.

Notwithstanding the robust methodological design, our findings must be interpreted in the context of several limitations. First and foremost, the statistical power of our MR analysis is constrained by the relatively small number of hypertensive encephalopathy cases (n=98) available in the FinnGen cohort. While the use of a large control group (n=374,631) mitigates some concerns, MR analyses with such a low case count are inherently underpowered to detect small to moderate causal effects and are more susceptible to inflated type I (false positive) or type II (false negative) errors. The resulting estimates, particularly the odds ratios and their confidence intervals, should be considered preliminary and interpreted with caution. Although our primary IVW results reached nominal statistical significance and were supported by consistent directional trends across sensitivity methods, these findings require replication in larger, independent cohorts with more HE cases to confirm their validity and improve precision. The statistical power of our analysis is inherently constrained by the limited number of hypertensive encephalopathy cases, potentially inflating the uncertainty of effect estimates. Although extensive sensitivity analyses suggested no detectable horizontal pleiotropy, its residual influence cannot be entirely ruled out. Furthermore, the exclusive reliance on European-ancestry summary statistics may restrict the generalizability of our results across diverse populations. Crucially, the estimated effect reflects lifelong genetic exposure to altered cytokine levels, which may not directly correspond to the consequences of acute pharmacological modulation. Finally, the pathophysiological complexity of hypertensive encephalopathy likely involves numerous inflammatory mediators beyond the five cytokines examined herein.

Although this study focuses on five specific cytokines with biological rationality and provides genetic evidence of their potential causal association with hypertensive encephalopathy, it is only a preliminary framework for understanding the role of the immune system in hypertensive encephalopathy. The inflammatory process in hypertension and its complications involves a vast and complex network of cytokines. As pointed out in the editor’s opinion, a broader study of the combination of inflammatory mediators will provide a more comprehensive understanding. Future MR studies can utilize an expanding database of quantitative trait loci (pQTL) in proteins to systematically scan dozens or even hundreds of circulating inflammatory proteins. This hypothesis-free approach helps to discover new and unexpected immune markers associated with the risk of hypertensive encephalopathy, distinguish core drivers from concomitant phenomena, and may reveal different endophenotypes of inflammation (for example, characterized by chemokine-driven, interleukin-driven or growth factor-driven). Integrating genomic, proteomic and clinical phenotypic data will be a key step in ultimately clarifying which inflammatory pathways are the true causal contributors to target organ damage in hypertension and which are merely associated markers. This will lay a solid foundation for the development of precise prevention and treatment strategies targeting specific inflammatory pathways.

## References

[1] van den Born BH, Lip GYH, Brguljan-Hitij J, Cremer A, Segura J, Morales E (2019). ESC council on hypertension position document on the management of hypertensive emergencies. Eur Heart J Cardiovasc Pharmacother.

[2] Zhang Z, Zhao L, Zhou X, Meng X, Zhou X (2022). Role of inflammation, immunity, and oxidative stress in hypertension: new insights and potential therapeutic targets. Front Immunol.

[3] Schetz M, De Jong A, Deane AM, Druml W, Hemelaar P, Pelosi P (2019). Obesity in the critically ill: a narrative review. Intensive Care Med.

[4] Takano T, Koyanagi A, Osawa Y, Taga T, Fujino H (2001). Cerebrospinal fluid interleukin-6 levels in hypertensive encephalopathy: a possible marker of disease activity. Ann Neurol.

[5] Zouein FA, Kurdi M, Booz GW (2013). LIF and the heart: just another brick in the wall?. Eur Cytokine Netw.

[6] Banner LR, Patterson PH, Allchorne A, Poole S, Woolf CJ (1998). Leukemia inhibitory factor is an anti-inflammatory and analgesic cytokine. J Neurosci.

[7] László A, Lénárt L, Illésy L, Fekete A, Nemcsik J (2019). The role of neurotrophins in psychopathology and cardiovascular diseases: psychosomatic connections. J Neural Transm.

[8] Merayo-Chalico J, Barrera-Vargas A, Juárez-Vega G, Alcocer-Varela J, Arauz A, Gómez-Martín D (2018). Differential serum cytokine profile in patients with systemic lupus erythematosus and posterior reversible encephalopathy syndrome. Clin Exp Immunol.

[9] Ronchetti S, Labombarda F, Roig P, De Nicola AF, Pietranera L (2023). Beneficial effects of the phytoestrogen genistein on hippocampal impairments of spontaneously hypertensive rats (SHR). J Neuroendocrinol.

[10] Davies NM, Holmes MV, Davey Smith G (2018). Reading Mendelian randomisation studies: a guide, glossary, and checklist for clinicians. Bmj.

[11] Dang M, Li Y, Zhao L, Li T, Lu Z, Lu J (2024). Causal association between particulate matter 2.5 and Alzheimer’s disease: a Mendelian randomization study. Front Public Health.

[12] Ahola-Olli AV, Würtz P, Havulinna AS, Aalto K, Pitkänen N, Lehtimäki T (2017). Genome-wide association study identifies 27 loci influencing concentrations of circulating cytokines and growth factors. Am J Hum Genet.

[13] Emdin CA, Khera AV, Kathiresan S (2017). Mendelian randomization. JAMA.

[14] Zhao JH, Stacey D, Eriksson N, Macdonald-Dunlop E, Hedman ÅK, Kalnapenkis A (2023). Genetics of circulating inflammatory proteins identifies drivers of immune-mediated disease risk and therapeutic targets. Nat Immunol.

[15] Kurki MI, Karjalainen J, Palta P, Sipilä TP, Kristiansson K, Donner KM (2023). FinnGen provides genetic insights from a well-phenotyped isolated population. Nature.

[16] Kamat MA, Blackshaw JA, Young R, Surendran P, Burgess S, Danesh J (2019). PhenoScanner V2: an expanded tool for searching human genotype-phenotype associations. Bioinformatics.

[17] Palmer TM, Lawlor DA, Harbord RM, Sheehan NA, Tobias JH, Timpson NJ (2012). Using multiple genetic variants as instrumental variables for modifiable risk factors. Stat Methods Med Res.

[18] Larsson SC, Burgess S (2022). Appraising the causal role of smoking in multiple diseases: a systematic review and meta-analysis of Mendelian randomization studies. EBioMedicine.

[19] Larsson SC, Traylor M, Malik R, Dichgans M, Burgess S, Markus HS (2017). Modifiable pathways in Alzheimer’s disease: mendelian randomisation analysis. Bmj.

[20] Yavorska OO, Burgess S (2017). MendelianRandomization: an R package for performing Mendelian randomization analyses using summarized data. Int J Epidemiol.

[21] Burgess S, Butterworth A, Thompson SG (2013). Mendelian randomization analysis with multiple genetic variants using summarized data. Genet Epidemiol.

[22] Greco MF, Minelli C, Sheehan NA, Thompson JR (2015). Detecting pleiotropy in Mendelian randomisation studies with summary data and a continuous outcome. Stat Med.

[23] Bowden J, Davey Smith G, Haycock PC, Burgess S (2016). Consistent estimation in Mendelian randomization with some invalid instruments using a weighted median estimator. Genet Epidemiol.

[24] Bowden J, Davey Smith G, Burgess S (2015). Mendelian randomization with invalid instruments: effect estimation and bias detection through egger regression. Int J Epidemiol.

[25] Verbanck M, Chen CY, Neale B, Do R (2018). Detection of widespread horizontal pleiotropy in causal relationships inferred from Mendelian randomization between complex traits and diseases. Nat Genet.

[26] Junttila IS (2018). Tuning the cytokine responses: an update on interleukin (IL)-4 and IL-13 receptor complexes. Front Immunol.

[27] Vargas-Rojas MI, Solleiro-Villavicencio H, Soto-Vega E (2016). Th1, Th2, Th17 and treg levels in umbilical cord blood in preeclampsia. J Matern Fetal Neonatal Med.

[28] Lei Y, Tang R, Xu J, Wang W, Zhang B, Liu J (2021). Applications of single-cell sequencing in cancer research: progress and perspectives. J Hematol Oncol.

[29] Chung CY, Yang JT, Kuo YC (2013). Polybutylcyanoacrylate nanoparticles for delivering hormone response element-conjugated neurotrophin-3 to the brain of intracerebral hemorrhagic rats. Biomaterials.

[30] Gao Q, Zhang Y (2021). CXCL11 signaling in the tumor microenvironment. Adv Exp Med Biol.

[31] Toshner M, Rothman A (2020). IL-6 in pulmonary hypertension: why novel is not always best. Eur Respir J.

[32] Ishibashi T, Inagaki T, Okazawa M, Yamagishi A, Ohta-Ogo K, Asano R (2024). IL-6/gp130 signaling in CD4(+) T cells drives the pathogenesis of pulmonary hypertension. Proc Natl Acad Sci U S A..

[33] Wang Y, Yang Q, Cheng Y, Gao M, Kuang L, Wang C (2018). Myosin heavy chain 10 (MYH10) gene silencing reduces cell migration and invasion in the glioma cell lines U251, T98G, and SHG44 by inhibiting the Wnt/β-Catenin pathway. Med Sci Monit.

[34] Mehaffey E, Majid DSA (2017). Tumor necrosis factor-α, kidney function, and hypertension. Am J Physiol Renal Physiol.

